# Supplementary figures and images for: Effectiveness of a school-based programme of animal-assisted humane education in Hong Kong for the promotion of social and emotional learning: A quasi-experimental pilot study
Source: PLoS One. 2021 Mar 19;16(3):e0249033. doi: 10.1371/journal.pone.0249033 (PMC7978358; doi:10.1371/journal.pone.0249033)

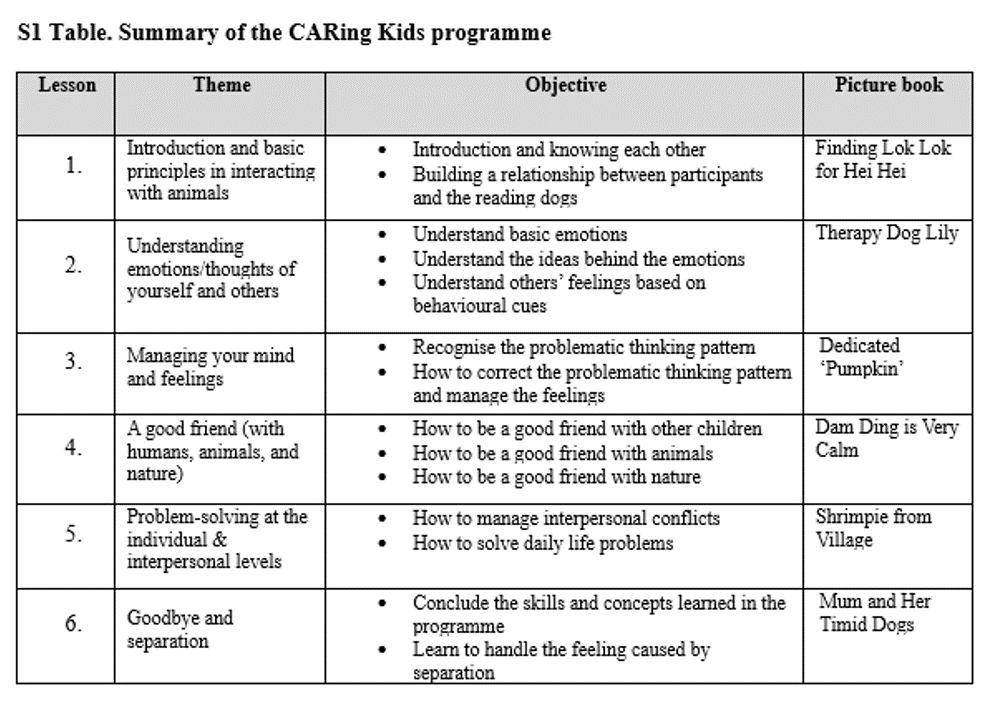

Supplement: S1 Table — (TIF) [file pone.0249033.s001.tif]
