# Supplementary material for: Effectiveness of a school-based programme of animal-assisted humane education in Hong Kong for the promotion of social and emotional learning: A quasi-experimental pilot study
Source: PLoS One. 2021 Mar 19;16(3):e0249033. doi: 10.1371/journal.pone.0249033 (PMC7978358; doi:10.1371/journal.pone.0249033)

Lesson 1
Introduction and Meeting the Reading Canines

Learning Objectives

- Understand the basic rules of the lessons
- Learn the basic principles in interacting with peers and animals (i.e., politeness and respect)
- Understand that animals and humans have feelings and thoughts
- Understand the feelings of animals and our peers

Role of Instructors

- The instructor is responsible for the implementation of the program, he/she needs to
  - Implement the program based on the teaching guidelines
  - Ensure the safety and welfare of both students and reading canines
  - Precautious to potential threats to any parties
    - Make sure that no harmful substance/food can be reached by participants/reading canines
    - Arrange the venue based on the sitting plan as stipulated in the Teachers' Manual
    - Be aware of the calming signals of the reading canines and avoid excess stressful situation
    - Clean the room before and after the session using animal-friendly detergents
- The handler is responsible to protect the welfare of the reading canines and to enhance the quality of student-animal interactions, he/she needs to
  - Notify the instructor if he/she finds that the reading canines is showing excessive stress signals
  - Facilitate the student-animal interaction during the reading to canine's activity

Materials

- Display cards on basic lesson rules
- Name Cards for the reading canines
- Storybooks "Finding Lok Lok for Hei Hei."*

Introduction (10 minutes)

<u>Activity: Introduce the lesson rules</u>

Scripts: "We are going to being the six-session CARing Kids Curriculums today. I want to introduce to you some special classmates. However, we need your cooperation to ensure that all of us are having a good time while we are learning. I wish you can follow these rules in class."

- Sit down and listen carefully
- Speak Soft

- Show respect to others/canines
- Do not grab the legs and tail of reading canines
- Be kind to others/dogs

How to greet the reading canines (10 minutes)

Activity 2: Introduce the reading canines and ways to greet them

Scripts: "To let our special classmates know who you are, we need to learn how to greet them politely. Here are some guidelines. I will show you how to greet them, please observe carefully. "(Teachers can invite a handler and his/her reading canines to demonstrate)

1. Approach the handler and introduce yourself
2. Seek handler's permission to pet the dog.
3. Slowly move to the side of the dog, adjust your body posture (stand up or squat down) based on the size of the dogs
4. Let the reading canines smell and sniff your hand
5. Praise and pet the reading canines gently

If the students are ready, the teacher can invite 1 to 2 students to try under the handler's supervision. The teacher should provide positive feedback if the student shows respect and politeness during the demonstration.

Interview the handler (10 minutes)

Activity: Getting to know your reading dog.

Scripts: "We have already greeted the reading canines; we are going to know more about them. Each group can ask the handler three questions about the reading canines. We would use the information you found for the next game."

The teacher invites three students in each group to ask the handler a question about the reading canines. The student needs to follow the below in asking a question.

1. Say your name
2. Look at the handler while asking
3. Say "Thank you" after the handler answer the questions

My new classmate (5 Minutes)

<u>Activity: Guess Who am I</u>

Script: "Now that we know more about our new classmates, I will now show you their name cards with some, but not all of their information. Can you match the name cards to the dog?"

The name card includes the following information:

1. Age
2. Sex
3. Personality
4. Favourite activities

Script: "Now we know the reading canines more, you might've <u>noticed that each dog has their interest, personality and story, like all of us</u>. "

Reading to dogs (35 minutes)

<u>Activity: Student reading to the reading canines</u>

Script: "Like all of us, every dog has their own story. I am going to invite you to read a part of the picture book to the reading canines."

The teacher divides the students into three to four groups (depends on the number of reading canines). The students can enter the reading zone to read to the reading canines. The handler can arrange the position of the students to ensure that they can comfortably interact the reading canines. Each group would read 3 to 4 pages of the story; then the teacher can ask questions with reference to the reading guide.

Discussion and Conclusion (5 minutes)

<u>Activity: Conclusion and Lesson Summary</u>

Script: "We all have our unique 'Name Card'. Both dogs and humans have their own story, feelings, thoughts, and experiences. Therefore, we need to respect others, including our peers and other non-human species. Let's summarize what we have learnt today."

1. Class rules
2. How to show your respect and politeness in social interaction
3. Both dogs and humans have their own emotions, experiences, and interests
4. Every life is unique and needs to be respected.

---

*Copyright of the story book is owned by publisher bbluesky: Hong Kong.

Figure 1.

The Classroom Arrangement of CARing Kids

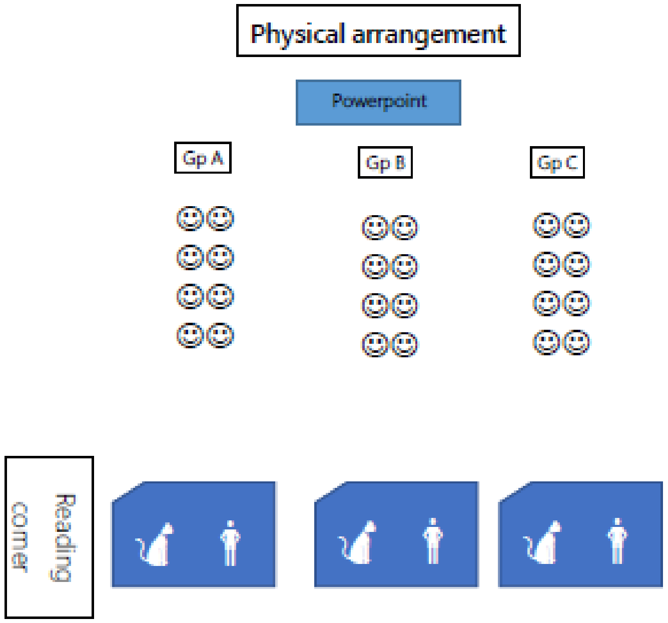

Supplement: S1 File — (PDF) [file pone.0249033.s002.pdf]
